# Concentration- and Temperature-Induced Phase Transitions in PrAlO_3_–SrTiO_3_ System

**DOI:** 10.1186/s11671-015-1225-4

**Published:** 2016-01-13

**Authors:** Leonid Vasylechko, Roman Stepchuk, Yuri Prots, Helge Rosner

**Affiliations:** Lviv Polytechnic National University, 12 Bandera Street, 79013 Lviv, Ukraine; Max-Planck-Institut für Chemische Physik fester Stoffe, Nöthnitzer Str. 40, 01187 Dresden, Germany

**Keywords:** Perovskite aluminates and titanates, Crystal structure, Solid solution, Phase transitions, 61, 61.05.cp, 64.70.K-

## Abstract

Single-phase mixed aluminates-titanates Pr_1−*x*_Sr_*x*_Al_1−*x*_Ti_*x*_O_3_ (*x* = 0.1, 0.2, 0.3, 0.5, 0.7) with rhombohedral perovskite structure were prepared by solid-state reaction technique at 1823–1873 K. Morphotropic rhombohedral-to-cubic phase transition in Pr_1−*x*_Sr_*x*_Al_1−*x*_Ti_*x*_O_3_ series is predicted to occur at *x* = 0.88. The temperature-induced structural phase transition *R* $\overline{3}$ *с* − *Pm*$\overline{3}$ *m* in Pr_0.5_Sr_0.5_Al_0.5_Ti_0.5_O_3_, detected at 930 K by in situ high-temperature X-ray synchrotron powder diffraction, occurs at considerably lower temperature as the corresponding transformation in the parent compound PrAlO_3_ (1770 K). Such remarkable drop of the transition temperature is explained by gradual decrease of the perovskite structure deformation in the Pr_1−*x*_Sr_*x*_Al_1−*x*_Ti_*x*_O_3_ series with increasing Sr and Ti contents as a consequence of the increasing Goldschmidt tolerance factor. For Pr_0.3_Sr_0.7_Al_0.3_Ti_0.7_O_3_ phase, a sequence of the low-temperature phase transformation *R* $\overline{3}$ *с − Immb*(*C*2/*m*) *− I*4/*mcm* was detected.

## Background

Rare earth aluminates *R*AlO_3_ with perovskite structure and SrTiO_3_-based materials show diverse technological application. In particular, they are used as solid electrolytes and anode materials in solid oxide fuel cells, as substrates for thin film epitaxy, materials for laser hosts, scintillates and phosphors, high-temperature ceramics and refractory materials ([1–5] and references herein). Due to the opposite signs of the temperature coefficient of the resonant frequency (*τ*_*f*_) of the *R*AlO_3_ and SrTiO_3_ compounds, mixed aluminates-titanates formed in the *R*AlO_3_–SrTiO_3_ systems are considered as prospective microwave materials with a high dielectric constant, moderate quality factor and a near zero value of *τ*_*f*_ [5–7].

During the last decade, *R*AlO_3_–SrTiO_3_ systems are of considerable interest in the physics of materials used in modern engineering. The two-dimensional electron gas at the interface between two insulators LaAlO_3_ and SrTiO_3_ [8] has been an active research area in the field-tunable metal-insulator transition, 2D superconductivity, coexistence of superconductivity and ferromagnetism, etc*.* [9–11]. Just recently, a similar effect was reported on the interfaces of SrTiO_3_ and *R*AlO_3_ (*R* = La, Pr, Nd) and *R*GaO_3_ compounds (*R* = La, Nd) in both crystalline and amorphous forms [12].

The aim of the present work is the study of the phase and structural behaviour of the mixed aluminates-titanates formed in the PrAlO_3_–SrTiO_3_ pseudo-binary system. At room temperature, the end members of the system—PrAlO_3_ and SrTiO_3_—adopt different variants of perovskite structure—rhombohedral *R*$\overline{3}$ *с* and cubic *Pm* $\overline{3}$ *m*, respectively. Rhombohedral PrAlO_3_ transforms into the cubic perovskite structure at about 1770 K ([4], and references herein). In addition, PrAlO_3_ undergoes a sequence of low-temperature (LT) structural phase transformations from the rhombohedral to an orthorhombic *Imma* structure at 205 K and from orthorhombic to a monoclinic *C*2/*m* structure at 151 K ([4], and references herein). Strontium titanate SrTiO_3_ undergoes a low-temperature structural phase transition from the cubic to the tetragonal *I*4*/mcm* perovskite structure below 105 K [13, 14]. Owing to the abovementioned peculiarities of the crystal structures PrAlO_3_ and SrTiO_3_ and their structural instabilities, extremely complex phase and structural behaviour are expected in the mixed praseodymium-strontium aluminate-titanate system.

## Methods

Mixed aluminates-titanates Pr_1−*x*_Sr_*x*_Al_1−*x*_Ti_*x*_O_3_ (*x* = 0.1, 0.2, 0.3, 0.5, 0.7) were prepared from stoichiometric amounts of the constituent oxides Pr_6_O_11_, Al_2_O_3_, TiO_2_ and strontium carbonate SrCO_3_ by solid-state reaction technique. The precursor powders were ball milled in ethanol for 3–6 h, dried, pressed in the pellets and sintered in air at 1673–1773 K for 18 h (the samples with *x* = 0.1 and 0.2) and at 1593 K for 24 h (the samples with *x* = 0.3, 0.5 and 0.7). After regrinding and powdering, the obtained products were pressed in the pellets and repeatedly fired in air at 1873 K (*x* = 0.1 and 0.2) and 1823 K (*x* = 0.3, 0.5 and 0.7) for 10 h.

X-ray powder diffraction technique (Huber imaging plate Guinier camera G670, Cu *K*α_1_ radiation) was used for the phase and structural characterization of the samples at room temperature. Thermal behaviour of the mixed aluminates-titanates has been studied exemplarily on Pr_0.5_Sr_0.5_Al_0.5_Ti_0.5_O_3_ and Pr_0.3_Sr_0.7_Al_0.3_Ti_0.7_O_3_ samples in the temperature ranges of 298–1173 K and 20–298 K, respectively. Corresponding *in situ* high-resolution X-ray synchrotron powder diffraction experiments were performed at beamlines B2 at HASYLAB/DESY (Hamburg, Germany) and ID22 at ESRF (Grenoble, France) during beamtimes allocated to the experiments I-20110214 and hc2044, respectively.

All crystallographic calculations including full-profile Rietveld refinement were performed by using WinCSD program package [15].

## Results and Discussion

**Fig. 1:**
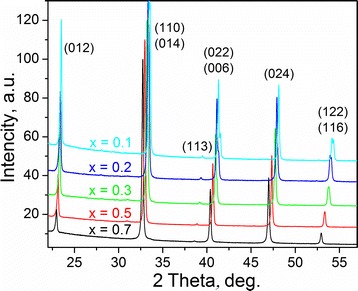
Experimental XRD patterns (Cu *K*α_1_ radiation, λ = 1.54056 Å) of the Pr_1-*x*_Sr_*x*_Al_1-*x*_Ti_*x*_O_3_ series

Examination of X-ray powder diffraction patterns revealed a formation of the single-phase perovskite structures in all samples synthesised (Fig. 1). No traces of parasitic phases were detected. An analysis of the splitting of diffraction maxima in the Pr_1−*x*_Sr_*x*_Al_1−*x*_Ti_*x*_O_3_ patterns with *x* = 0.1, 0.2, 0.3 and 0.5 proves rhombohedral deformation of the perovskite structure. The rhombohedral splitting decreases with the increase in the content of strontium and titanium in the Pr_1−*x*_Sr_*x*_Al_1−*x*_Ti_*x*_O_3_ series, completely vanishing in the Pr_0.3_Sr_0.7_Al_0.3_Ti_0.7_O_3_ sample. However, the presence of a weak (113) reflection at the diffraction pattern of this sample (Fig. 1) indicates that its structure still remains rhombohedral.

**Fig. 2:**
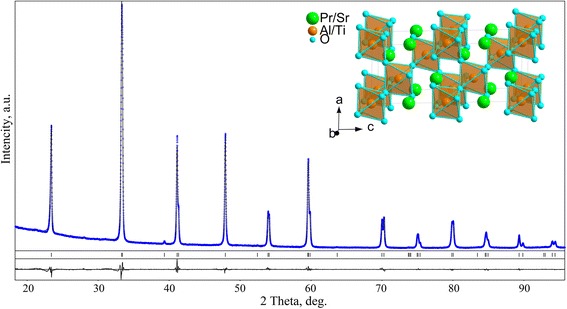
Graphical results of Rietveld refinement of Pr_0.8_Sr_0.2_Al_0.8_Ti_0.2_O_3_. The experimental XRD pattern (*blue dots*) is shown in comparison with the calculated one. *Vertical bars* indicate positions of diffraction maxima in space group *R* $\overline{3}$ *с*. The *inset* shows the view of the structure as corner-shared Al/TiO_6_ octahedra with Pr/Sr species located between them

From the experimental XRD patterns of Pr_1−*x*_Sr_*x*_Al_1−*x*_Ti_*x*_O_3_ samples, the crystal structure parameters of the mixed aluminates-titanates were derived. Full-profile Rietveld refinement performed in space group *R* $\overline{3}$ *с* resulted in excellent agreement between calculated and experimental profiles (see, for example, Fig. 2) and led to the final structural parameters and residuals presented in Table 1.

**Table 1:** Unit cell parameters, coordinates and isotropic displacement parameters of atoms in Pr_1-*x*_Sr_*x*_Al_1-*x*_Ti_*x*_O_3_ structures at RT

Atoms, sites	Parameters, residuals	*x* in Pr_1−*x*_Sr_*x*_Al_1−*x*_Ti_*x*_O_3_				
		0.1	0.2	0.3	0.5	0.7
	*a*, Å	5.35081(8)	5.37046 (4)	5.38979 (4)	5.42806 (5)	5.4614 (3)
	*c*, Å	13.0303 (3)	13.0868 (2)	13.1442 (2)	13.2569 (2)	13.378 (2)
Pr/Sr, 6*c* (0, 0, ¼)	*B*_iso_, Å^2^	0.88 (2)	0.69 (2)	0.85 (1)	0.95 (2)	0.67 (2)
Al/Ti, 6*b* (0, 0, 0)	*B*_iso_, Å^2^	0.78 (7)	0.70 (5)	0.78 (3)	0.67 (3)	0.43 (3)
O, 18*e* (*x*, 0, ¼)	*x*	0.5485 (14)	0.5429 (6)	0.5364 (7)	0.5333 (8)	0.5239 (11)
	*B*_iso_, Å^2^	2.00 (15)	1.12 (6)	1.37 (6)	1.49 (7)	1.22 (7)
	*R*_*I*_	0.070	0.048	0.041	0.026	0.029
	*R*_*P*_	0.089	0.056	0.064	0.066	0.059

**Fig. 3:**
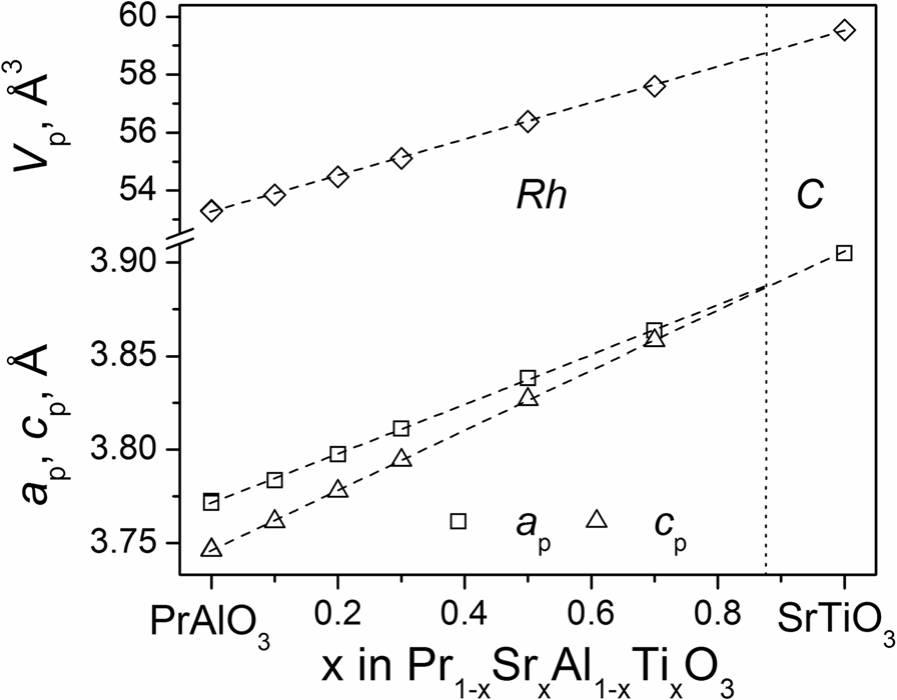
Unit cell dimensions of Pr_1-*x*_Sr_*x*_Al_1-*x*_Ti_*x*_O_3_ series. The rhombohedral lattice parameters are normalized to the perovskite cell as follows: *a*_*p*_ *= a*_*r*_/√2, *c*_*p*_ *= c*_*r*_/√12, *V*_*p*_ *= V*_*r*_ */6*. The *dashed line* marks the phase boundary between the *Rh* and the *C* phases

Comparison of the obtained structural parameters of the praseodymium-strontium mixed aluminates-titanates with the literature data for the “pure” PrAlO_3_ and SrTiO_3_ (Fig. 3) clearly proves the formation of the extended solid solution Pr_1−*x*_Sr_*x*_Al_1−*x*_Ti_*x*_O_3_ with rhombohedral perovskite structure. A morphotropic phase transition from rhombohedral to the cubic perovskite structure in the Pr_1−*x*_Sr_*x*_Al_1−*x*_Ti_*x*_O_3_ series can be expected at *x* = 0.88, as it follows from the analysis of the concentration dependence of the unit cell dimensions of the rhombohedral lattice (Fig. 3). In the related LaAlO_3_–SrTiO_3_ system, the rhombohedral solid solution exists up to 60 mole % of LaAlO_3_; after that, the transition to the cubic perovskite structure takes place [16].

**Fig. 4:**
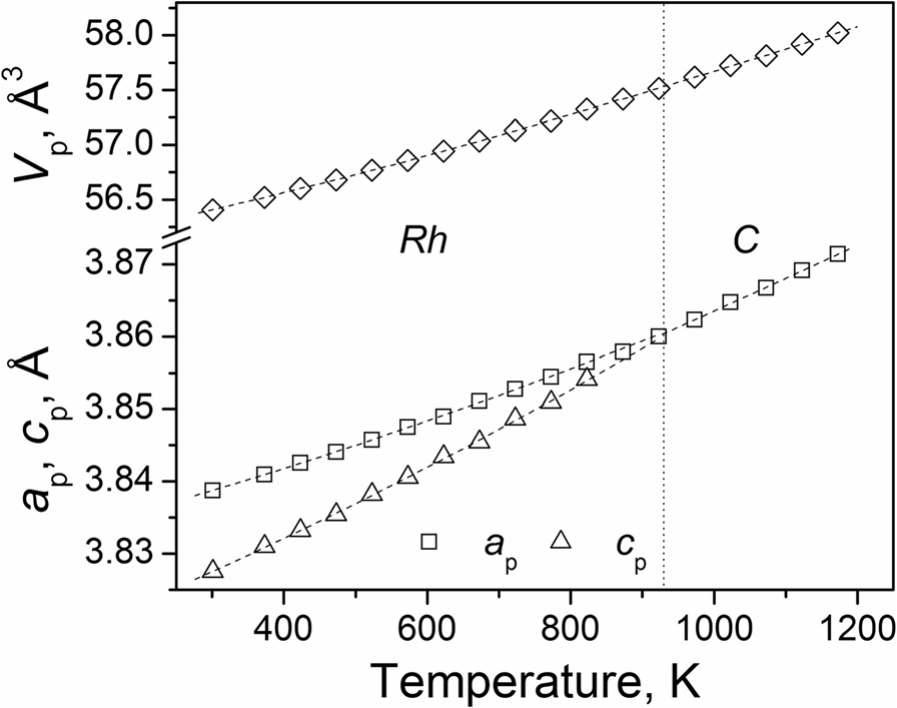
Temperature evolution of unit cell dimensions of Pr_0.5_Sr_0.5_Al_0.5_Ti_0.5_O_3_. The rhombohedral lattice parameters are normalized to the perovskite cell as follows: *a*_*p*_ *= a*_*r*_/√2, *c*_*p*_ *= c*_*r*_/√12, *V*_*p*_ *= V*_*r*_ */6*. The *dashed line* marks the phase boundary between the *Rh* and the *C* phases

*In situ* high-temperature X-ray synchrotron powder diffraction investigation of the Pr_0.5_Sr_0.5_Al_0.5_Ti_0.5_O_3_ sample revealed a continuous phase transition from rhombohedral to the cubic perovskite structure at elevated temperatures. As it was established from the temperature-resolved X-ray synchrotron powder diffraction measurements, the rhombohedral lattice parameters *a* and *c* increase anisotropically with temperature and merge together at 930 K, when the transition to the ideal perovskite structure occurs (Fig. 4).

In comparison with the parent PrAlO_3_ compound, in which transformation to the cubic perovskite structure occurs around 1770 K [4], the *R* $\overline{3}$ *с − Pm* $\overline{3}$ *m* transition in Pr_0.5_Sr_0.5_Al_0.5_Ti_0.5_O_3_ takes place at considerably lower temperature of 930 K. Such remarkable drop of the phase transition temperature can be explained by gradual decrease of the perovskite structure deformation in the Pr_1−*x*_Sr_*x*_Al_1−*x*_Ti_*x*_O_3_ series with increasing of Sr and Ti content. According to the phase diagram of the *R*AlO_3_-based perovskite systems [4], the temperature of the *R* $\overline{3}$ *с − Pm* $\overline{3}$ *m* phase transition decreases linearly with increasing radii of *R* cation as a consequence of the increasing Goldschmidt tolerance factor.

**Fig. 5:**
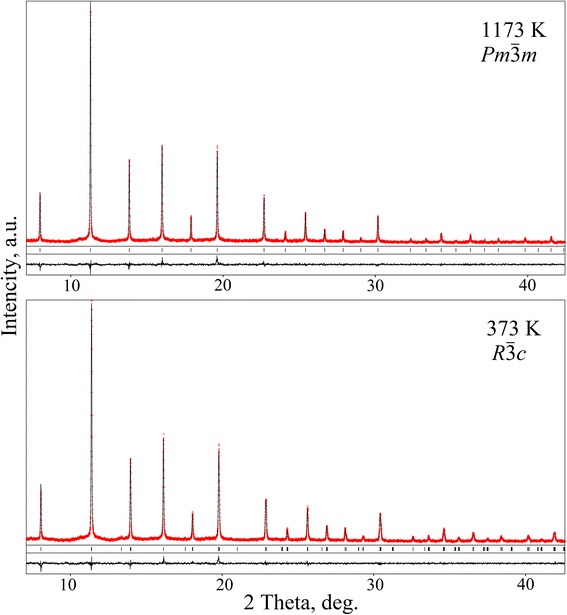
X-ray synchrotron powder diffraction patterns of Pr_0.5_Sr_0.5_Al_0.5_Ti_0.5_O_3_ (HASYLAB B2 data, *λ* = 0.53820 Å). Full-profile Rietveld refinement was performed in space groups *Pm* $\overline{3}$ *m* (T = 1173 K) and *R* $\overline{3}$ *с* (T = 373 K). Experimental (*dots*) and calculated patterns, difference profiles and positions of the diffraction maxima are given

Graphical results of Rietveld refinement of the high-temperature modifications of the Pr_0.5_Sr_0.5_Al_0.5_Ti_0.5_O_3_ structure and refined structural parameters at selected temperatures are presented in Fig. 5 and Table 2, respectively.

**Table 2:** Refined structural parameters of rhombohedral and cubic modifications of Pr_0.5_Sr_0.5_Al_0.5_Ti_0.5_O_3_

Atoms, sites	Parameters, residuals	Temperature, space group					
		373 K	573 K	823 K	923 K	1073 K	1173 K
		*R* $\overline{3}$ *с*	*R* $\overline{3}$ *с*	*R* $\overline{3}$ *с*	*Pm* $\overline{3}$ *m*	*Pm* $\overline{3}$ *m*	*Pm* $\overline{3}$ *m*
	*a*, Å	5.4319 (2)	5.4412 (2)	5.4541 (5)	3.8600 (2)	3.8667 (2)	3.8714 (2)
	*c*, Å	13.2708 (7)	13.3041 (9)	13.350 (2)	-	-	-
Pr/Sr, 6*c* in *R* $\overline{3}$ *с*; 1*b* in *Pm* $\overline{3}$ *m*	*x*	0	0	0	½	½	½
	*y*	0	0	0	½	½	½
	*z*	¼	¼	¼	½	½	½
	*B*_iso_, Å^2^	1.15 (3)	1.35 (3)	1.69 (3)	1.92 (5)	2.01 (5)	2.19 (5)
Al/Ti, 6*b* in *R* $\overline{3}$ *с*; 1*a* in *Pm* $\overline{3}$ *m*	*x*	0	0	0	0	0	0
	*y*	0	0	0	0	0	0
	*z*	0	0	0	0	0	0
	*B*_iso_, Å^2^	0.72 (5)	0.92 (6)	0.95 (6)	0.85 (8)	1.19 (9)	1.05 (9)
O, 18*e* in *R* $\overline{3}$ *с*; 3*d* in *Pm* $\overline{3}$ *m*	*x*	0.530 (3)	0.529 (3)	0.506 (16)	½	½	½
	*y*	0	0	0	0	0	0
	*z*	¼	¼	¼	0	0	0
	*B*_iso_, Å^2^	0.7 (2)	0.8 (2)	1.9 (2)	2.9 (2)	2.9 (2)	3.5 (2)
	*R*_*I*_	0.047	0.048	0.041	0.045	0.047	0.046
	*R*_*P*_	0.147	0.145	0.064	0.128	0.133	0.135

**Fig. 6:**
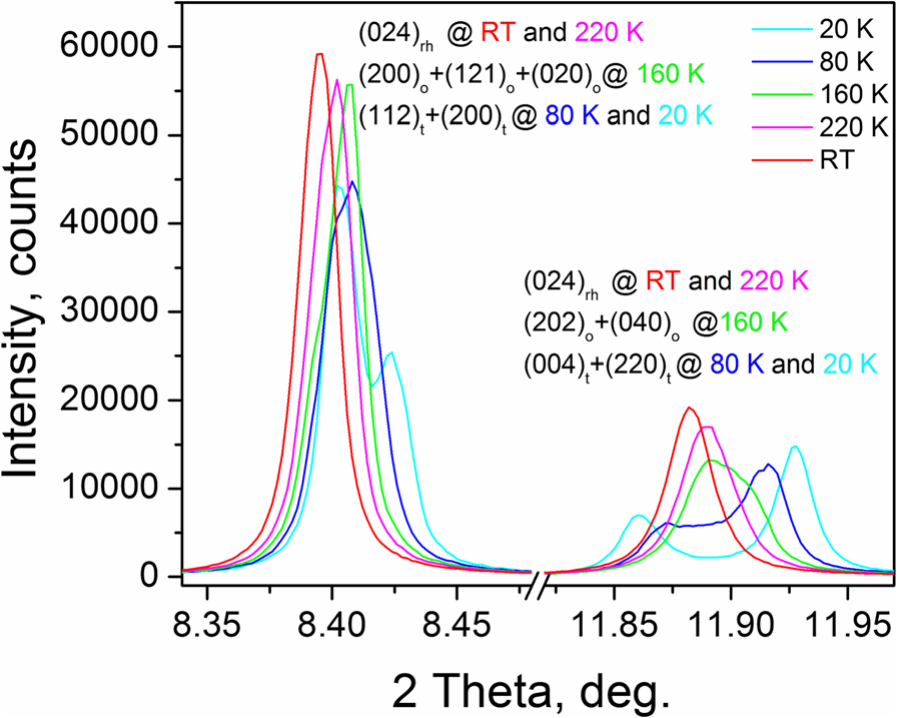
Fragments of LT synchrotron powder diffraction patterns of Pr_0.3_Sr_0.7_Al_0.3_Ti_0.3_O_3_ (ESRF ID22 data, *λ* = 0.40003 Å). Splitting of (110)_*c*_ and (200)_*c*_ reflections in rhombohedral (rh), orthorhombic (o) and tetragonal (t) modifications of Pr_0.3_Sr_0.7_Al_0.3_Ti_0.3_O_3_ is shown

Spot-check examination of low-temperature structural behaviour of the Pr_1−*x*_Sr_*x*_Al_1−*x*_Ti_*x*_O_3_ system was performed on the example of a Pr_0.3_Sr_0.7_Al_0.3_Ti_0.3_O_3_ sample at temperatures of 20, 80, 160 and 220 K. Extremely high resolution of the beamline ID22 at ESRF allows to detect subtle changes in the reflections splitting at different temperature measurements (Fig. 6), which clearly prove a sequence of LT phase transformations in this sample.

Crystal structures of Pr_0.3_Sr_0.7_Al_0.3_Ti_0.3_O_3_ at RT and at 220 K were refined in the space group *R* $\overline{3}$ *с*, thus confirming the results derived from the convenient XRD data (Table 1). Taking into account the character of the reflection splitting, crystal structure parameters of the low-temperature modification of Pr_0.3_Sr_0.7_Al_0.3_Ti_0.3_O_3_ at 20 and 80 K were successfully refined in space group *I*4/*mcm*. X-ray synchrotron diffraction features of Pr_0.3_Sr_0.7_Al_0.3_Ti_0.3_O_3_ at 160 K could be successfully modelled either in the orthorhombic *Immb* or in the monoclinic *I*2/*m* (*C*2/*m*) perovskite structure. Since in both cases during the refinement procedures, practically the same residuals were obtained, a preference should be given to the more symmetric orthorhombic structure. Taking into account that the end members of the system show different sequences of LT phase transformations *R* $\overline{3}$ *с − Immb − C*2/*m* (PrAlO_3_) and *Pm* $\overline{3}$ *m − I*4/*mcm* (SrTiO_3_), additional investigations are required in order to shade light on the complex phase and structural behaviour of the mixed aluminates-titanates below RT.

## Conclusions

The formation of extended solid solution Pr_1−*x*_Sr_*x*_Al_1−*x*_Ti_*x*_O_3_ with rhombohedral perovskite structure has been revealed in the PrAlO_3_–SrTiO_3_ pseudo-binary system based on X-ray powder diffraction data. An analysis of the obtained structural parameters in comparison with the data for the parent compounds PrAlO_3_ and SrTiO_3_ revealed a decrease of perovskite structure deformation in Pr_1−*x*_Sr_*x*_Al_1−*x*_Ti_*x*_O_3_ series with increasing Sr and Ti content as a consequence of the increasing Goldschmidt tolerance factor. As a result, concentration-induced phase transition from a rhombohedral to the cubic perovskite structure takes place in the Pr_1−*x*_Sr_*x*_Al_1−*x*_Ti_*x*_O_3_ system at *x* = 0.88. A decreasing structure deformation in Pr_1−*x*_Sr_*x*_Al_1−*x*_Ti_*x*_O_3_ series leads to the significant decrease of the temperature-induced phase transition *R* $\overline{3}$ *с − Pm* $\overline{3}$ *m* from 1770 to 930 K in PrAlO_3_ and Pr_0.5_Sr_0.5_Al_0.5_Ti_0.5_O_3_, respectively. The sequence of the low-temperature phase transition *R* $\overline{3}$ *с − Immb*(*I*2/*m*) *− I*4/*mcm* in Pr_0.3_Sr_0.7_Al_0.3_Ti_0.3_O_3_ revealed during the spot-check X-ray synchrotron powder diffraction examination promises an extremely complex picture of the phase and structural relations in PrAlO_3_–SrTiO_3_ system below room temperature. Further structural and calorimetric investigations of the system are in progress.
